# Passage Number-Induced Replicative Senescence Modulates the Endothelial Cell Response to Protein-Bound Uremic Toxins

**DOI:** 10.3390/toxins13100738

**Published:** 2021-10-19

**Authors:** Fatima Guerrero, Andres Carmona, Maria Jose Jimenez, Teresa Obrero, Victoria Pulido, Juan Antonio Moreno, Sagrario Soriano, Alejandro Martín-Malo, Pedro Aljama

**Affiliations:** 1 Maimonides Biomedical Research Institute of Cordoba (IMIBIC), Reina Sofia University Hospital, University of Cordoba, 14004 Córdoba, Spain; andres.carmona@imibic.org (A.C.); mariajose.jimenez@imibic.org (M.J.J.); teresa.obrero@imibic.org (T.O.); victoria.pulido@imibic.org (V.P.); marias.soriano.sspa@juntadeandalucia.es (S.S.); amartinma@senefro.org (A.M.-M.); paljamag@gmail.com (P.A.); 2 Department of Medicine, University of Cordoba, 14004 Córdoba, Spain; 3 Department of Cell Biology, Physiology and Immunology, University of Cordoba, 14014 Córdoba, Spain; 4 Nephrology Unit, Reina Sofia University Hospital, 14004 Córdoba, Spain; 5 Spanish Renal Research Network, Institute of Health Carlos III, 28040 Madrid, Spain

**Keywords:** aging, indoxyl sulfate, p-cresol, endothelial cells, endothelial dysfunction, senescence

## Abstract

Endothelial aging may be induced early in pathological situations. The uremic toxins indoxyl sulfate (IS) and p-cresol (PC) accumulate in the plasma of chronic kidney disease (CKD) patients, causing accelerated endothelial aging, increased cardiovascular events and mortality. However, the mechanisms by which uremic toxins exert their deleterious effects on endothelial aging are not yet fully known. Thus, the aim of the present study is to determine the effects of IS and PC on endothelial damage and early senescence in cultured human umbilical vein endothelial cells (HUVECs). Hence, we establish an in vitro model of endothelial damage mediated by different passages of HUVECs and stimulated with different concentrations of IS and PC to evaluate functional effects on the vascular endothelium. We observe that cell passage-induced senescence is associated with apoptosis, ROS production and decreased endothelial proliferative capacity. Similarly, we observe that IS and PC cause premature aging in a dose-dependent manner, altering HUVECs’ regenerative capacity, and decreasing their cell migration and potential to form vascular structures in vitro. In conclusion, IS and PC cause accelerated aging in HUVECs, thus contributing to endothelial dysfunction associated with CKD progression.

## 1. Introduction

Endothelial aging may be triggered by different mechanisms, such as increased apoptosis, oxidative stress and deleterious effects on cell proliferation [1]. These senescence-associated factors promote structural and functional changes in the vasculature that contribute to the development and progression of cardiovascular disease (CVD) [2]. Patients with chronic kidney disease (CKD) show an accelerated or premature aging process, which is characterized by a persistent microinflammatory status known as “inflammaging” [3,4]. This premature aging may be associated to the increased presence of protein-bound uremic toxins in the plasma of CKD patients, such as indoxyl sulfate (IS) and p-cresol (PC). Both toxins have deleterious effects on the body, affecting different cellular and tissue functions [5]. Furthermore, these uremic toxins have been associated with the development of cardiovascular events and increased cardiovascular mortality in CKD patients, and they are implicated in the progression of renal disease [6,7]. Protein-bound uremic toxins are difficult to be removed during dialysis because of their high binding affinity to serum albumin [8,9,10]. Currently, many different strategies for removing protein-bound uremic toxins from blood have been applied. However, these adjustments have not carry out successful outcomes [11].

In vitro, cellular senescence is a process that occurs after a certain number of cell cycles and/or in response to excessive stressors [12,13]. Senescent cells may affect a number of physiological functions, thus contributing to aging and the development of chronic diseases [14]. Uremic serum activates endothelial cells in vitro [15]. Uremic toxins, especially protein-bound toxins, are likely pathogenic agents inducing endothelial dysfunction in CKD [16,17]. In vitro models of endothelial damage induced by the uremic toxins IS and PC are commonly used. However, the conditions used, particularly the concentration of IS or PC, vary widely [18,19,20,21,22,23,24,25,26]. On the other hand, most of the studies were carried out in young endothelial cells, and to date, there is no evidence that this model has been performed in senescent endothelial cells. Furthermore, the mechanisms by which uremic toxins exert their deleterious effects on endothelial aging are not yet fully known. Previous studies reported that uremic toxins showed harmful effects on endothelial cells; however, these studies did not analyze toxic effects in correlation with cell age. For this reason, we evaluated the effects of different concentrations of indoxyl sulfate and p-cresol on apoptosis, oxidative stress and endothelial proliferation at different passage numbers. Additionally, this study focused on determining the effects of indoxyl sulfate and p-cresol on endothelial damage and early senescence in cultured human umbilical vein endothelial cells (HUVECs).

## 2. Results

### 2.1. Vascular Senescence Determines Apoptosis, Reactive Oxygen Species (ROS) and Proliferation Rate in Cultured HUVECs

**Figure 1:**
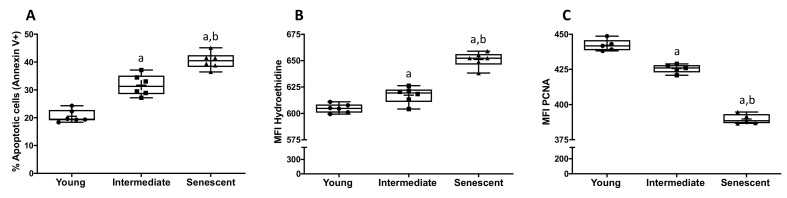
Replicative senescence modulates apoptosis, ROS and proliferative rate in endothelial cells (HUVECs). The apoptosis rate (**A**) was quantified by flow cytometry, and the results are expressed as the percentage of annexin V-positive cells. ROS production (**B**) and cell proliferation capacity (**C**) were also quantified by flow cytometry, and data are expressed as the mean fluorescent intensity (MFI) of hydroethidine (**B**) and proliferating cell nuclear antigen (PCNA) (**C**). Data are depicted as the median (Q1, Q3) of at least five independent experiments. The bar extending from the boxes indicates variability outside the upper and lower quartiles. The mean is represented as (+). a: *p* < 0.01 vs. Young HUVECs; b: *p* < 0.01 vs. Intermediate HUVECs.

Aging alters the functional properties of the vascular endothelium. For this reason, we developed an in vitro approach to analyze several parameters associated with vascular aging in HUVECs. In serial subcultures, we determined the apoptosis rate, ROS production and proliferation capacity of young (2–9 passages), intermediate (15–25 passages) and senescent HUVECs (>30 passages). As shown in Figure 1A, the number of apoptotic cells increased significantly and progressively according to the cell passages (20.73 ± 1.12, 31.69 ± 1.55 and 40.48 ± 1.20 in young, intermediate and senescent HUVECs, respectively, *p* < 0.01).

Likewise, intracellular ROS production significantly increased in senescent cells (651.05 ± 2.91) as compared to both intermediate (617.29 ± 3.15, *p* < 0.01) and young cells (604.77 ± 1.72, *p* < 0.01). At the same time, intermediate HUVECs exhibited a significant increase in intracellular ROS compared to early passage cells (*p* = 0.01) (Figure 1B). As it is reported in Figure 1C, cell proliferation capacity decreased in senescent cells (389.66 ± 1.56) as compared with intermediate (425.63 ± 1.35, *p* < 0.01) and young HUVECs (442.17 ± 1.84, *p* < 0.01). Furthermore, we observed a significant decrease in cell proliferation capacity between intermediate and young HUVECs (*p* < 0.01).

### 2.2. Effects of Indoxyl Sulfate during Replicative Senescence

**Figure 2:**
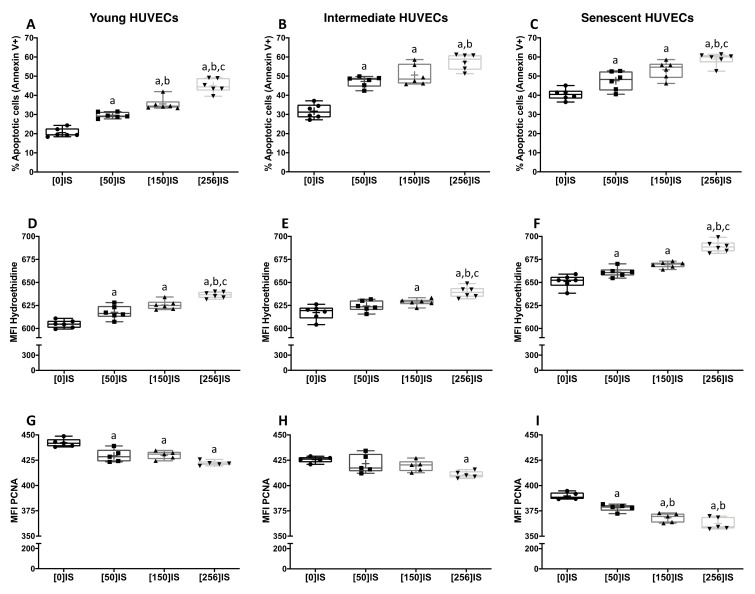
Indoxyl sulfate (IS) induces apoptosis, oxidative stress and a decrease in proliferation in endothelial cells (HUVECs) during replicative senescence. HUVECs from various passages were exposed to different concentrations of IS (0–256 µg/mL) for 24 h. The apoptosis rate (**A**–**C**) was quantified by flow cytometry, and the results are expressed as the percentage of annexin V-positive cells. ROS production (**D**–**F**) and cell proliferation capacity (**G**–**I**) were also quantified by flow cytometry, and data are expressed as the mean fluorescent intensity (MFI) of hydroethidine (**D**–**F**) and PCNA (**G**–**I**). Data are depicted as the median (Q1, Q3) of at least five independent experiments. The bar extending from the boxes indicates variability outside the upper and lower quartiles. The mean is represented as (+). a: *p* < 0.05 vs. control cells; b: *p* < 0.05 vs. IS (50 µg/mL); c: *p* < 0.05 vs. IS (150 µg/mL).

We then evaluated the effect of different uremic toxins in our experimental model. IS increased apoptosis in a dose-dependent manner, irrespectively of cell age (Figure 2A–C). Significant differences were observed between groups treated with 50 and 150 µg/mL of IS in young HUVECs.

Similarly, in a dose-dependent manner, IS-treated HUVECs showed significantly higher ROS levels when compared to controls (Figure 2D–F). In intermediate cells, doses of IS above 150 µg/mL increased oxidative stress with respect to the untreated cells (Figure 2E). The proliferative capacity of HUVECs was impaired after IS administration in a dose-dependent manner (Figure 2G–I). In young (Figure 2G) and senescent HUVECs (Figure 2I), a progressive but non-significant decrease in proliferation was detected after stimulation with different doses of IS. On the other hand, no significant changes were noted in intermediate HUVECs treated with the lowest dose of IS as compared to the control (Figure 2H).

Intergroup differences were observed in apoptosis, ROS and PCNA between aged and young endothelial cells after stimulation with the different doses of IS (*p* < 0.02).

### 2.3. Effects of P-Cresol during Replicative Senescence

**Figure 3:**
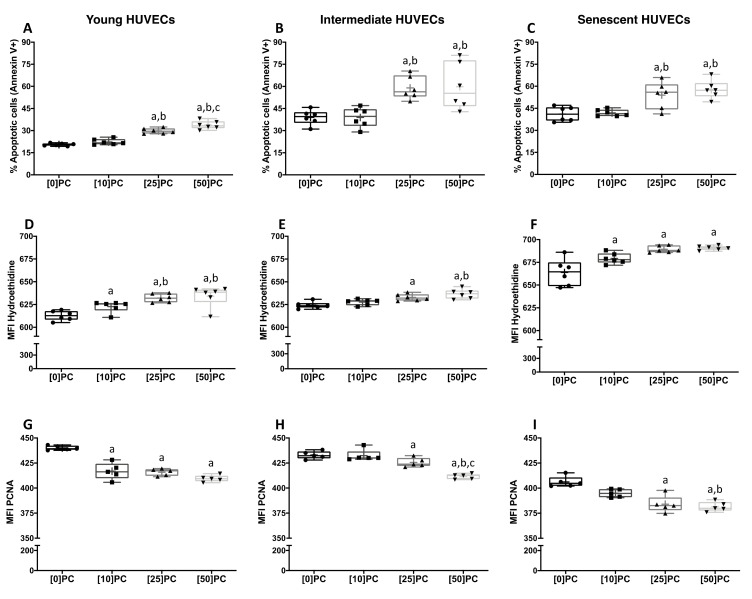
P-cresol (PC) induces apoptosis, oxidative stress and a decrease in proliferation in endothelial cells (HUVECs) during replicative senescence. HUVECs from various passages were exposed to different concentrations of PC (0–50 µg/mL) for 24 h. The apoptosis rate (**A**–**C**) was quantified by flow cytometry, and the results are expressed as the percentage of annexin V-positive cells. ROS production (**D**–**F**) and cell proliferation capacity (**G**–**I**) were also quantified by flow cytometry, and data are expressed as the mean fluorescent intensity (MFI) of hydroethidine (**D**–**F**) and PCNA (**G**–**I**). Data are depicted as the median (Q1, Q3) of at least five independent experiments. The bar extending from the boxes indicates variability outside the upper and lower quartiles. The mean is represented as (+). a: *p* < 0.05 vs. control cells; b: *p* < 0.05 vs. PC (10 µg/mL); c: *p* < 0.05 vs. PC (25 µg/mL).

An increased number of apoptotic cells were observed in young HUVECs treated with PC (25–50 µg/mL) as compared to the untreated HUVECs, but no differences were found between both concentrations (Figure 3A). Similar results were observed in intermediate (Figure 3B) and senescent cells (Figure 3C), although the data were less homogeneous compared to young cells. Increased intercellular ROS production was observed after treatment with different PC concentrations in young HUVECs (Figure 3D). In intermediate HUVECs, PC only induced ROS production at higher doses (Figure 3E). By contrast, in senescent cells, the low dose of PC was enough to increase ROS production (Figure 3F). PC also decreased the proliferative capacity of HUVECs. A progressive but non-significant decrease was detected in young HUVECs at different doses of PC (Figure 3G). Moreover, in intermediate cells, doses above 25 µg/mL of PC were needed to find a statistically significant difference with respect to the untreated cells (Figure 3H). By contrast, in senescent cells, low concentrations of PC were enough to significantly decrease the levels of PCNA (Figure 3I).

Intergroup differences were detected in apoptosis, ROS and PCNA between aged and young endothelial cells after stimulation with the different concentrations of PC (*p* < 0.05).

### 2.4. Indoxyl Sulfate and P-Cresol Induce Accelerated Senescence in Young HUVECs

As it is reported in the previous figures, young HUVECs treated with IS or PC showed similar numbers of apoptotic cells to those found in aged HUVECs without uremic toxins. The same results were observed when we analyzed intracellular ROS production or PCNA levels. Thus, young cells exposed to IS and PC showed increased ROS production and a lower proliferation capacity than untreated aged HUVECs.

### 2.5. Uremic Toxins Induce Early Senescence in Young HUVECs

**Figure 4:**
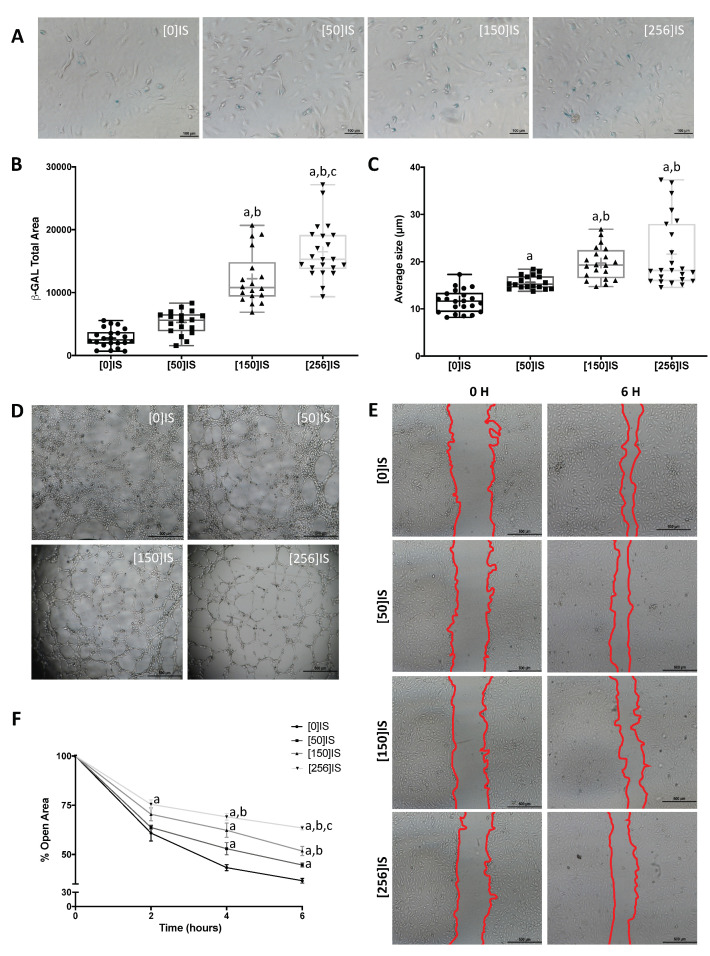
Indoxyl sulfate (IS) promoted early senescence and endothelial dysfunction. (**A**) Representative images of inverted optical microscopy of HUVECs stimulated with several concentrations of IS (0–256 μg/mL). (**B**) β-gal staining levels in HUVECs across all experimental conditions. (**C**) Average size (µm) of β-gal-positive cells. Results are expressed as the median (Q1, Q3) of at least five independent experiments. a: *p* < 0.01 vs. control; b: *p* < 0.01 vs. IS (50 μg/mL); c: *p* < 0.05 vs. IS (150 μg/mL). (**D**) Representative images of tube-like three-dimensional structures of HUVECs on a semi-natural matrix, Matrigel, 2 h after seeding. (**E**) Representative images of the wound test in HUVECs stimulated with different IS concentration (red lines show the extent of the wound). (**F**) Data points represent the percent of open area. Results are shown as means ± SEM of at least five different experiments. a: *p* < 0.05 vs. control; b: *p* < 0.05 vs. IS (50 μg/mL); c: *p* < 0.01 vs. IS (150 μg/mL).

**Figure 5:**
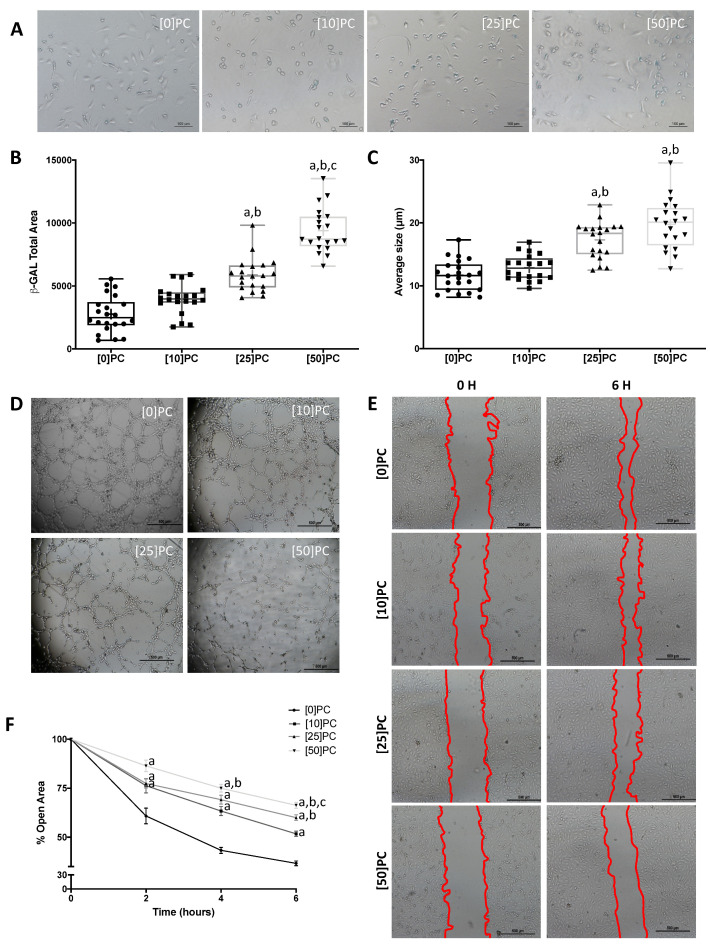
P-cresol (PC) promoted early senescence and endothelial dysfunction. (**A**) Representative images of inverted optical microscopy of HUVECs stimulated with several concentrations of PC (0–50 μg/mL). (**B**) β-gal staining levels in HUVECs across all experimental conditions. (**C**) Average size (µm) of β-gal-positive cells. Results are expressed as the median (Q1, Q3) of at least five independent experiments. a: *p* < 0.01 vs. control; b: *p* < 0.01 vs. PC (10 μg/mL); c: *p* < 0.01 vs. PC (25 μg/mL). (**D**) Representative images of tube-like three-dimensional structures of HUVECs on a semi-natural matrix, Matrigel, 2 h after seeding. (**E**) Representative images of the wound test in HUVECs stimulated with different PC concentration (red lines show the extent of the wound). (**F**) Data points represent the percent of open area. Results are shown as means ± SEM of at least five different experiments. a: *p* < 0.05 vs. control; b: *p* < 0.05 PC (10 μg/mL); c: *p* < 0.05 vs. PC (25 μg/mL).

Increased senescence was observed in HUVECs treated with uremic toxins, as demonstrated with the β-galactosidase assay (Figure 4A,B and Figure 5A,B). Thus, the total β-galactosidase-labeled area (Figure 4B and Figure 5B) and average size (Figure 4C and Figure 5C) were higher in HUVECs treated with IS and PC.

### 2.6. Uremic Toxins Promote Endothelial Dysfunction in Young HUVECs

As shown in Figure 4D and Figure 5D, HUVECs treated with the uremic solutes showed a reduced capacity to form new vascular vessels. In the same line, we also found a decreased number (Nb) of master junctions, a reduced Nb of segments and a lower Nb of branches of vessels in young HUVECs stimulated with IS or PC, independently of the dose used (Table 1 and Table 2). However, total tube length values decreased in a dose-dependent manner in both IS and PC solutes (Table 1 and Table 2).

On the other hand, six hours after the wound was performed, we found a dose-dependent reduction in endothelial proliferation after exposure to IS or PC (Figure 4E and Figure 5E). IS at 50, 150 and 250 µg/mL decreased wound healing by 18% (*p* < 0.05), 29% (*p* < 0.01) and 42% (*p* < 0.01), respectively (Figure 4F). In the same way, the treatment with PC at 10, 25 and 50 µg/mL reduced cell migration by 29% (*p* < 0.01), 39% (*p* < 0.01) and 45% (*p* < 0.01), respectively (Figure 5F).

## 3. Discussion

This is the first study showing the effect of uremic toxins on apoptosis, oxidative stress and proliferation during replicative senescence. We have demonstrated that the uremic toxins IS and PC cause accelerated aging in HUVECs, with an increased apoptosis rate, ROS production and decreased endothelial proliferative capacity. Moreover, IS and PC alter the regenerative capacity of HUVECs, decreasing cell migration and the potential to form vascular structures. Our results also indicate that early passage HUVECs exposed to uremic toxins are similar to non-stimulated aged HUVECs.

Aging is currently considered one of the most important cardiovascular risk factors [27,28]. A number of molecular and functional alterations are observed in cells during the aging process, leading to the development and progression of disease [29,30]. It is known that endothelial aging may be induced early in pathological situations. This endothelial aging promotes the development of endothelial dysfunction and consequently CVD [31,32]. The severity of endothelial dysfunction appears to increase as senescence is enhanced. In this sense, there is accumulated data supporting the contribution of cellular senescence to vascular injury in CKD [33,34,35,36]. This fact would explain the elevated incidence of CVD in the elderly or in patients suffering from accelerated endothelial aging.

Cells undergoing replicative senescence acquire characteristics of aging cells [30]. Senescent endothelial cells show enhanced β-galactosidase activity, G0/G1 phase arrest, abnormal proliferative capacity, increased apoptosis and ROS production [37,38,39]. Consistent with these data, we observed that increased cell passages induced senescence in HUVECs, as demonstrated by the enhanced apoptosis rate, ROS production and reduced endothelial proliferative capacity. These functional changes may contribute to vascular deterioration associated with aging.

Young or middle-aged CKD patients show premature endothelial aging [33,40]. Endothelial senescence in CKD patients is the consequence of multiple mediators [41]. One of these factors may be related to the fact that the endothelium of CKD patients is constantly exposed to numerous uremic toxins, such as IS and PC, causing cellular stress and cell damage [1,42]. Protein-bound uremic toxins have been associated with progression of CKD, cardiovascular disease and high mortality in uremic patients [6,43,44]. The European Uremic Toxin Work Group (EUTox) provides data on uremic toxins serum concentrations in the uremic population [44,45]. In uremic patients the mean IS or PC concentration reported is 53.0 ± 91.5 and 20.1 ± 10.5 mg/L respectively, with a maximum concentration of 236.0 mg/L for IS and 40.7 mg/L for PC. According to this information, we have used a concentration ranges similar to those found in CKD patients, from the low reported uremic concentration to the highest one.

The role of uremic toxins in vascular dysfunction has been extensively investigated [1,42]. However, there are contradictory data. Some authors reported that uremic toxins promoted apoptosis, ROS generation and cell cycle arrest in endothelial cells [25,26,46,47,48]. Contrary to these articles, other researchers did not find differences after uremic toxin exposure [46,47]. Differences in the concentrations of uremic toxins employed may explain these discrepancies. Interestingly, most of the studies were carried out in early passage cells. Since aging may influence these pathogenic effects, we evaluated the effect of uremic toxins during replicative senescence. Our data indicate a progressive increase in senescence-related characteristics (apoptosis, ROS production and loss of proliferative potential) in HUVECs treated with IS or PC. Curiously, we observed that senescent cells are still inducible by external stimuli. These inducible senescent cells could accumulate in the vascular endothelium, creating an adverse microenvironment that would trigger endothelial malfunction. This could justify, in part, why elderly people with CKD are more likely to develop vascular disease [49,50] and endothelial senescence [34,51].

In this study, we have also demonstrated that uremic toxins can accelerate the appearance of a senescent phenotype not only in elderly HUVECs but also in early passage cells [52,53,54]. Our data indicate that early passage HUVECs exposed to uremic toxins share common features with cells in replicative senescence. This phenomenon is evidenced because IS- or PC-treated young HUVECs showed similar apoptosis rates, ROS production and proliferative capacities to non-stimulated aged HUVECs. Previous studies have reported that uremic toxins caused adverse effects on mitochondrial function, decreased anti-aging defenses and induced inflammation and cellular senescence, thus promoting a pro-aging milieu which favors vascular dysfunction [2,34,42,55]. In this sense, our results show that uremic toxins can alter the functional capacity of endothelial cells to regenerate and migrate, as well as their angiogenic ability in vitro. Taken together, uremic toxin-induced oxidative stress could be promoting aging processes and therefore contributing to endothelial cell dysfunction.

## 4. Conclusions

This study provides relevant information about the effects of endothelial aging on apoptosis, proliferation and oxidative stress. These processes are modulated by indoxyl sulfate and p-cresol during replicative senescence. Moreover, senescent endothelial cells are inducible by uremic toxins, amplifying endothelial damage in the senescent microenvironment. Finally, our data prove that uremic toxins induce a phenotypic differentiation of young HUVECs toward senescent cells, thus promoting endothelial dysfunction.

## 5. Materials and Methods

### 5.1. Cell Culture

Human umbilical vein endothelial cells (HUVECs, Cell Systems (Clonetics, Solingen, Germany)) were cultured at 37 °C in 95% humidity and a 5% CO_2_ atmosphere. HUVECs were cultured in endothelial cell basal medium (EBM) plus endothelial cell growth medium supplements (EGM, Cambrex Bioscience, Walkersville, MD, USA) and 10% fetal bovine serum (FBS) (Invitrogen, Carlsbad, CA, USA). Briefly, fresh cells were seeded at a density of 2500 to 3000 cell/cm^2^ in T-75 flasks (Falcon). The medium was changed every 48 h. By light microscopic examination we observed that cultures reached confluence after 6–7 days.

According to previous group experiments [56], cells passaged 2–9 times were defined as young, cells passaged 15–25 times were defined as intermediate and those passaged >30 times were considered as senescent.

Each HUVEC type (young, intermediate or senescent) was stimulated for 24 h with different concentrations of p-cresol (10, 25 and 50 µg/mL) or indoxyl sulfate (50, 150 and 250 µg/mL) to determine the apoptosis rate, oxidative stress and cell proliferation. All assays were quantified and analyzed by flow cytometry on a FACSCalibur cytometer (Becton Dickinson Biosciences (BD); San Jose, CA, USA) equipped with standard CellQuest software. Data were collected for 10,000 cells per sample.

### 5.2. Preparation of Uremic Toxins

The uremic toxins used in this study were indoxyl sulfate potassium salt and p-cresol 4-methylphenol (Sigma-Aldrich, St. Louis, MO, USA). Indoxyl sulfate was dissolved in water to obtain a stock solution of 0.397 M. Working solutions were prepared by a 1:10 (*v*/*v*) dilution of the stock solution with culture medium. P-cresol was diluted with methanol to a final concentration of 0.0956 M for use.

### 5.3. Apoptosis Assay

The cell apoptosis rate was assessed with the annexin V staining kit (BD) according to the manufacturer’s recommendations. Briefly, the cells were resuspended in 1X binding buffer (10 mM Hepes/NaOH, 140 mM NaCl, 5 mM CaCL_2_, pH 7.4) at a concentration of 1 × 10^6^ cells/mL. An amount of 5 μL annexin V-PE was added to 100 μL of the cell suspension and incubated for 15 min in the dark at room temperature (RT). Subsequently, cells were washed with phosphate-buffered saline and finally diluted with 500 μL of binding buffer. The percentage of apoptosis was analyzed by flow cytometry. Negative tube controls contained annexin V binding buffer.

### 5.4. Detection of Reactive Oxygen Species (ROS) or Hydroethidine

Hydroethidine (HE) (Invitrogen) was used to determine HUVECs’ oxidative stress in vitro. Hydroethidine, also called dihydroethidium, exhibits blue fluorescence in the cytosol until being oxidized by ROS to become ethidium and emit a red color. Cells were exposed to HE (2 μM) for 15 min in darkness at room temperature (RT). Then, cells were washed with PBS and fixed with BD CellFIX (BD). Intracellular superoxide levels or intracellular ROS production were quantified and analyzed by flow cytometry.

### 5.5. Cell Proliferation

HUVEC proliferation was evaluated by proliferating cell nuclear antigen (PCNA) labeling using a kit containing PE-PCNA antibody (BD). In brief, HUVECs were permeabilized using FACS Permeabilizing Solution (BD). After vortexing, cells were incubated for 10 min at RT in the dark. Then, cells were washed with PBS and incubated with 20 μL of anti-PCNA antibody for 20 min at RT in the dark. The cells were washed with PBS and fixed with CellFIX (BD). PCNA levels were quantified and analyzed by flow cytometry.

### 5.6. Senescence Assay

Senescence–Galactosidase Staining Kit (MBL International Corporation; Woburn, MA, USA) was used to detect senescent cells. Cells were fixed and washed twice with PBS. After that, staining solution was added and cells were incubated overnight at 37 °C without CO_2_. Afterward, senescence-associated beta-galactosidase-positive cells (senescent cells) were identified as blue-stained cells using an inverted microscope (OPTIKA Microscopes, Ponteranica, Italy) in three–five random fields for each well and quantified with the ImageJ software.

### 5.7. Matrigel Tube Formation Assay

To evaluate the effect of uremic toxins on vascular network formation, an in vitro angiogenesis assay was performed. Fifteen-well plates (IBIDI, GmbH, Gräfelfing, Germany) were coated with 10 µL of Matrigel (Corning; Steuben, NY, USA) using a cool pipette to avoid the formation of bubbles and incubated at 37 °C for 30 min in 5% CO_2_. Endothelial cells were trypsinized, and a total of 10^4^ cells/well were plated onto Matrigel for their angiogenic activity. In parallel, 10% FBS was administered as an internal positive control. After 2 h, photographs were taken with an optical inverted microscope (OPTIKA Microscopes) and quantified with the ImageJ software. Results were expressed as previously described by Izuta et al. [57].

### 5.8. Wound Healing Assay

In the wound assay, each well of the culture plate was scratched across the center using a sterile micropipette tip. Afterward, the cells were washed with PBS to eliminate the cellular debris detached from the “wound” zone. Next, culture medium was added, and HUVECs were incubated at 37 °C and 5% CO_2_. The healing process was monitored from 2 to 6 h. At the indicated intervals, photographs were taken with an optical inverted microscope (OPTIKA Microscopes), and an automated analysis was performed with the ImageJ software. Cell migration was determined by the rate of cells moving towards the scratched area.

### 5.9. Statistical Analysis

Data distribution was analyzed with the Shapiro–Wilk test to verify a normal distribution. Comparisons between means were analyzed by ANOVA with Bonferroni post hoc correction. Non-parametric data were analyzed by the Mann–Whitney U test or the Kruskal–Wallis test. Values of *p* lower than 0.05 were considered to be significant. Data were analyzed using SPSS Statistics software version 25.0 (SPSS, Inc., Chicago, IL, USA) or GraphPad Prism 6.0c (GraphPad Software, La Jolla, CA, USA). Data are depicted in box and whisker plots (min to max).

## Figures and Tables

**Table 1:** Angiogenic parameters in HUVECs treated with indoxyl sulfate (IS). Tube formation was analyzed in HUVECs by measurement of the number of master junctions, number of segments, number of branches and total length after treatment with different concentrations of IS. Data are the means ± SEM of at least five independent experiments. ^a^ *p* < 0.05 vs. control; ^b^ *p* < 0.05 vs. IS (50 μg/mL); ^c^ *p* < 0.05 vs. IS (150 μg/mL).

IS Treatment (μg/mL)	Number of Master Junctions	Number of Segments	Number of Branches	Total Length
0	137.5 ± 9.8	462.8 ± 38.4	108.8 ± 6.2	22,881 ± 647.1
50	106.5 ± 8.9 ^a^	253 ± 14.9 ^a^	89.7 ± 6.1	20,224 ± 622 ^a^
150	82.8 ± 2.7 ^a^	223 ± 13.5 ^a^	71.3 ± 2.5 ^a^	17,875 ± 348.5 ^a,b^
256	60.2 ± 3.1 ^a,b^	146.2 ± 5.6 ^a,b,c^	64.1 ± 3.9 ^a,b^	13,404 ± 596.9 ^a,b,c^

**Table 2:** Angiogenic parameters in HUVECs treated with p-cresol (PC). Tube formation was evaluated by measurement of the number of master junctions, number of segments, number of branches and total length after treatment with different concentrations of PC. Data are the means ± SEM of at least five independent experiments. ^a^ *p* < 0.05 vs. control; ^b^ *p* < 0.05 vs. PC (10μg/mL); ^c^ *p* < 0.05 vs. PC (25 μg/mL).

PC Treatment (μg/mL)	Number of Master Junctions	Number of Segments	Number of Branches	Total Length
0	137.5 ± 9.8	462.8 ± 38.4	108.8 ± 6.2	22,881 ± 647.1
10	98 ± 3.2 ^a^	280 ± 11.4 ^a^	77.2 ± 2.7 ^a^	19,688 ± 351.1 ^a^
25	70.8 ± 6.1 ^a^	204 ± 14.3 ^a^	65.8 ± 5.2 ^a^	16,988 ± 477.7 ^a,b^
50	57.5 ± 4.5 ^a,b^	162 ± 13.54 ^a,b^	56.4 ± 4.9 ^a^	14,470 ± 975.2 ^a,b,c^

## Data Availability

The authors confirm that the data supporting the findings of this study are contained within the manuscript. Raw data are available from the corresponding author, Guerrero F, upon reasonable request.
